# The EBRAINS Hodgkin-Huxley Neuron Builder: An online resource for building data-driven neuron models

**DOI:** 10.3389/fninf.2022.991609

**Published:** 2022-09-26

**Authors:** Luca Leonardo Bologna, Roberto Smiriglia, Carmen Alina Lupascu, Shailesh Appukuttan, Andrew P. Davison, Genrich Ivaska, Jean-Denis Courcol, Michele Migliore

**Affiliations:** ^1^Institute of Biophysics, National Research Council, Palermo, Italy; ^2^Centre National de la Recherche Scientifique, Institut des Neurosciences Paris-Saclay, Université Paris-Saclay, Saclay, France; ^3^Blue Brain Project, École Polytechnique Fédérale de Lausanne, Geneva, Switzerland

**Keywords:** data-driven brain models, online resources, EBRAINS, neuron, model optimization, high-performance computing

## Abstract

In the last decades, brain modeling has been established as a fundamental tool for understanding neural mechanisms and information processing in individual cells and circuits at different scales of observation. Building data-driven brain models requires the availability of experimental data and analysis tools as well as neural simulation environments and, often, large scale computing facilities. All these components are rarely found in a comprehensive framework and usually require *ad hoc* programming. To address this, we developed the EBRAINS Hodgkin-Huxley Neuron Builder (HHNB), a web resource for building single cell neural models via the extraction of activity features from electrophysiological traces, the optimization of the model parameters via a genetic algorithm executed on high performance computing facilities and the simulation of the optimized model in an interactive framework. Thanks to its inherent characteristics, the HHNB facilitates the data-driven model building workflow and its reproducibility, hence fostering a collaborative approach to brain modeling.

## Introduction

Computational neuroscience is now a well-established and powerful approach for: (1) Understanding neural mechanisms at different spatial and temporal scales; (2) inferring on neural dynamics that are not yet accessible via experimental measures and (3) making predictions able to suggest new experimental protocols and paradigms (Dayan and Abbott, 2001; Koch and Segev, 2001; Gerstner and Kistler, 2002; Van Ooyen, 2003). Neural models mirror the dynamics of individual cells and neural ensembles (Izhikevich, 2003, 2004; Bazenkov et al., 2020), small- and large-scale circuits and tissues (Samsonovich and Ascoli, 2005; Izhikevich and Edelman, 2008; Markram et al., 2011) and help to answer, or at least address, scientific questions concerning synaptic plasticity (Babadi and Abbott, 2013; Cortes et al., 2013), the role of spike timing and firing rate in neural activity (Brette, 2015), the stimulus-response dynamics (Pospischil et al., 2011), and individual cells and circuit functions (Migliore et al., 2018; Egger et al., 2020).

Depending on the scientific principles underlying the model construction, different levels of neural details can be implemented and, to cope with the wide range of computational neuroscientists’ needs, a number of simulation environments are already available, such as NEURON (Hines and Carnevale, 1997), GENESIS (Bower and Beeman, 2007), Brian (Goodman and Brette, 2008; Stimberg et al., 2019) and NEST (Gewaltig and Diesmann, 2007; Eppler, 2008), to cite a few. Also, modelers can rely on a growing ensemble of neural datasets, built in the framework of large-scale brain initiatives (Insel et al., 2013; Kandel et al., 2013; Amunts et al., 2016), to be leveraged for the fine tuning of the model parameters. In addition, high-performance computing facilities are already available for running highly detailed and/or large-scale models or model parameter search algorithms, such as the NeuroScience Gateway,^1^ the Swiss National Supercomputing Center,^2^ the Juelich Supercomputing Centre,^3^ the CINECA consortium.^4^

With the aim of providing the scientific community with open access and collaborative environments, several online platforms have been created. For example, ModelDB (McDougal et al., 2015, 2017), which has been actively maintained over the last three decades, have become the *de facto* standard for neural model sharing and archiving. The OpenSourceBrain promises to be a reference portal for computational neuroscientists, since it provides an online resource for the visualization, sharing, analysis and simulation of neural models standardized thanks to the NeuroML (Gleeson et al., 2010) and PyNN (Davison, 2008) model description languages. The number of platforms created for neuroscience data sharing is also increasing, e.g., DANDI Archive,^5^ Zenodo,^6^ Dryad,^7^ Figshare,^8^ Hippocampome.org,^9^ NeuroMorpho.Org^10^ and a number of initiatives foster the development of tools for collaborative data visualization and analysis, such as Neurodata Without Borders (Teeters et al., 2015) and the Allen Institute for Brain Science,^11^ which, in addition to a large dataset of neuroscience images and data, provides a dedicated Software Development Kit (SDK) for data analysis. Also the EBRAINS Knowledge Graph (KG), built in the framework of the Human Brain Project (HBP) (Amunts et al., 2016) and the EBRAINS research infrastructure, aims at providing a comprehensive ensemble of neuroscience data, models and tools. In addition, the EBRAINS Live Papers allow direct access to digital resources associated with scientific papers, which can be downloaded, visualized and, more generally, exploited via public EBRAINS tools and services (Appukuttan et al., 2022a).

Notwithstanding the availability of the above-mentioned tools, environments, and platforms, to the best of our knowledge, an integrated and comprehensive framework for the realization of a complete and efficient model building workflow that includes the model construction, the data-driven parameter optimization and a user-friendly interface for model visualization and running is not available to the scientific community. In an attempt to bridge this gap, we developed the EBRAINS Hodgkin-Huxley Neuron Builder (HHNB), a web-based resource that guides the user through the complete process of building a single cell NEURON model. Since the seminal papers of Hodgkin and Huxley, 1952a,b, introducing the set of non-linear ordinary differential equations governing the interplay among the different ion channels expressed on the neuronal membrane, any data-driven, biophysically accurate, computational model of neurons and networks is built upon these equations. This type of models have proven to be fundamental for understanding the subtle dynamics and predict new experimentally testable mechanisms underlying the behaviors of individual neurons and neural cell ensembles (Meunier and Segev, 2002; Catterall et al., 2012).

The HHNB allows the creation of such models and their optimization against experimental data, in research works where data-driven brain modeling is fundamental for understanding or predicting subtle neural dynamics, such as the investigation of the ion channel (Migliore et al., 2018), excitability (Vitale et al., 2021) and synaptic transmission (Hunt et al., 2022) properties in specific brain regions. It includes: (1) The extraction or upload of the electrophysiological features against which the model parameters are optimized; (2) the selection or creation of the NEURON model that will undergo the optimization process; (3) the use of HPC platforms for the optimal parameters search and (4) the simulation of the optimized model.

Thanks to the integration of several web resources and software packages and to its user-friendly nature, the HHNB provides the scientific community with unique functionalities such as: A fully interactive online interface for the visualization and selection of experimental traces from which the electrophysiological features of interest are extracted and used in the optimization process; an online editor for the construction/selection of a biophysically detailed single cell model and an online simulation environment where the simulation parameters can be set and the simulated activity downloaded; a seamless access to the HPC systems where the model optimization processes are run. Finally, by accessing the EBRAINS KG database, which is continuously updated with new models and neuroscience data, and storing the optimized models in the EBRAINS Model Catalog (Appukuttan et al., 2022b), the HHNB provides a unique resource for building and test biophysically detailed neural models following a collaborative approach.

## Methods

### Hodgkin-Huxley Neuron Builder frontend and backend

The EBRAINS HHNB is implemented via a Python-based Django project^12^ consisting of two Django applications, the *efelg*, which implements the EBRAINS NeuroFeatureExtract (NFE) web application and provides the algorithms and interface for the feature extraction procedures in the HHNB workflows and the *hh-neuron-builder*, which implements the frontend and backend for the model selection/upload, HPC job configuration, optimized model fetching, running and integration with the Model Catalog (see the Table 1). To guarantee seamless and fast communication and data management between the two web apps, they are hosted on the same Ubuntu 20.04 Virtual Machine (VM), in the CINECA supercomputer center (see Table 1). The CINECA VM consists of 60 GB RAM, 8 VirtualCPUs (VCPU) and is accessible via Openstack.^13^ The frontend has been developed in HTML, Javascript and CSS code and the backend in Python; frontend and backend interact via the Django routing system and users’ requests, performed via the HHNB GUI, are handled via the HTTPS protocol and managed by an NGINX web server coupled with a uWSGI web interface.

**TABLE 1:** List of software resources adopted in the paper, with references to publications (if available) and urls.

Software resources			

Name	References	Url	Usage in the HHNB
BluePyEfe	N/A	https://github.com/BlueBrain/BluePyEfe	Feature extraction workflow (eFEL Python wrapper)
BluePyOpt	Van Geit et al., 2016	https://github.com/BlueBrain/BluePyOpt	Single cell model optimization on HPC systems
CINECA	N/A	https://www.cineca.it/	Hosting of the Virtual Machines where the HHNB and the Service Account Utility are installed
CSCS	N/A	https://www.cscs.ch/	HPC system where the optimizations are run
EBRAINS Model Catalog	N/A	https://model-catalog.brainsimulation.eu/	Hosting of the single cell models chosen by the users for optimization
EBRAINS Hodgkin-Huxley Neuron Builder	This paper	https://github.com/ebrains-cls-interactive/hbp-bsp-hh-neuron-builder	N/A
EBRAINS NeuroFeatureExtract	Bologna et al., 2021	https://github.com/ebrains-cls-interactive/hbp-bsp-hh-neuron-builder/tree/master/efelg	Feature extraction workflow (web interface)
eFEL	N/A	https://github.com/BlueBrain/eFEL	Feature extraction workflow (core package)
NEURON	Carnevale and Hines, 2006	https://www.neuron.yale.edu	Model building
Neuroscience Gateway	Sivagnanam et al., 2013	http://www.nsgportal.org	HPC system where the optimizations are run
Service Account Utility	N/A	https://github.com/ebrains-cls-interactive/eb-clsi-service-account	Optimization job submission on behalf of users with no credentials on HPC systems

Requests from different users are handled asynchronously and independently via the creation and management of user-dedicated folder trees based on the file system and system resources. A system user with limited access and privileges to the VM resources performs the HHNB system operation, in order to limit security vulnerabilities. The code is publicly available (Gleeson et al., 2017) on GitHub, under the LGPLv3 license (see Table 1) and the use of the application is free of charge.

### Electrophys Feature Extraction Library and Blue Brain Python E-feature extraction library

The feature extraction functionalities of the HHNB are available to the users via the integration of the NFE web application (Bologna et al., 2021) in the HHNB GUI. This tool leverages the Electrophys Feature Extraction Library (eFEL) and Blue Brain Python E-feature extraction (BluePyEfe) libraries (see Table 1) that provide the core feature extraction software package and its convenient Python wrapper, respectively. More specifically, the eFEL accepts electrophysiological traces as input and extracts several activity features from the time signals. The features available for extraction belong to three different categories, depending on their properties (i.e., spike event features, spike shape features and voltage features) and can be selected via the HHNB interface. While the feature extraction code is optimized for the analysis of individual traces in the eFEL, higher level operations like feature grouping (e.g., by stimulus amplitude) and averaging are not implemented. For this reason, we integrated, in the HHNB backend, the BluePyEfe software library that provides a wrapper around the eFEL and allows to read different file formats, group the electrophysiological traces by cell and stimulus amplitude, average the feature values by cell and again by ensemble of cells. Finally, the feature extraction output files are appropriately formatted for the Blue Brain Python Optimization Library (BluePyOpt) (see Table 1) optimizer and are transparently integrated in the file package to be sent to the HPC system for the model optimization. For more details on the integration of the eFEL and BluePyEfe packages in the HHNB/NFE, see (Bologna et al., 2021), where the features of both libraries have been thoroughly discussed.

### Blue Brain Python Optimization Library

The model optimization process is carried out via the BluePyOpt, which is open source software implementing multi-objective model parameter optimization based on a genetic evolutionary algorithm (Van Geit et al., 2016). The latter implements the evolution of a population of parameters through consecutive generations. For each iteration a set of offspring individuals is generated from selected parents (following the principles of the genetic algorithm) belonging to the previous generation. Each individual contains a set of passive properties and peak ion channel consistent with the electrophysiological trace features provided. The cost function of the process is a score defined by the total error associated with each individual and calculated as the sum of the absolute deviations of the features observed in the simulated activity from its experimental counterpart.

In terms of implementation, while the BluePyOpt library is agnostic with respect to the model folder and file organization, the models in the dataset (and the model files the users can upload) are organized following a specific folder structure. The *mechanisms* folder contains the NEURON (see Table 1). *mod* files that describe the kinetics of the ion channels (these files contain instructions formalized in the NMODL language). The *morphology* folder contains the .*asc* file describing the cell morphology. These files are ASCII-encoded text files handled by the Neurolucida neuron reconstruction software. They contain tree-structured text building blocks appropriately formatted to represent the neural morphology in terms of compartment coordinates in the 3D space. Neuron somas are represented as contours named “cellbody.” The *config* folder contains: (1) The *features.json* file (describing the feature values used in the optimization process); (2) the *protocols.json* (describing the somatic current injection stimuli protocol applied to the experimental traces used in the feature extraction step); (3) the *parameters.json* file (reporting the model ion channels inserted in the morphology compartments, their distribution in the relative compartments, the constant parameters—the resting potential, the temperature, the capacitance of the membrane and the reversal potential of *Na* and *K*—and the list of conductances to be optimized for each compartment together with their minimum and maximum values, range and distribution); (4) the *morph.json* file, which indicates the morphology file name. Finally, the *model* folder contains the *analysis.py*, *evaluator.py*, and *template.py* files used for launching the optimization process on the HPC systems and performing the model activity analysis on the optimized model after the optimization process has been finalized. Any of the models available in the HHNB can be downloaded, should the user need an example of a structured model file.

BluePyOpt has been installed on CSCS HPC and NSG systems (see Table 1) using Spack,^14^ a package management tool designed to support multiple versions and configurations of software packages on a wide variety of platforms and environments. The software resides in dedicated folders where software packages are indexed and must be imported/loaded in the submitted configuration files executed once the job is launched.

### Service account utility

A service account is generally defined as a user account with special privileges on a specific system, which is used by the account owner (a physical person, legally responsible for the account usage) to submit jobs on behalf of third parties. The Service Account Utility we developed and integrated in the HHNB consists of a backend application implemented through the Django REST framework^15^ and running on a dedicated VM (hosted on the CINECA supercomputer center) and available to the developers through a set of APIs allowing to request a job submission to the CSCS-DAINT system (see Table 1). More specifically, to submit jobs (or perform other operations like results fetching or job listing) on the HPC supercomputers via the service account, users need to login to the EBRAINS authentication system via the HHNB GUI in order for the application to recognize them as EBRAINS account holders. Once the login has been completed, a token is generated that unequivocally identifies each user. This token is sent to the Service Account Utility server together with every request and its validity is verified before the operation is authorized. In the backend, once a request is received, the token is extrapolated and used for accounting operations needed to keep track of the time of job submission, allocated quota, job status, etc., in a local PostgreSQL database.

## Usage

### Overview

The HHNB is a full-stack web-application for data-driven optimization of single cell neural models. The driving principle of the HHNB is to provide the scientific community with a user-friendly online tool that allows optimization of the equation parameters of a biophysically detailed single neuron model, implemented in the NEURON simulation environment (Hines and Carnevale, 1997), with respect to experimental data, in order to increase the accuracy of its electrophysiological behavior and the plausibility of its biophysical details (see section “Model selection”).

**FIGURE 1:**
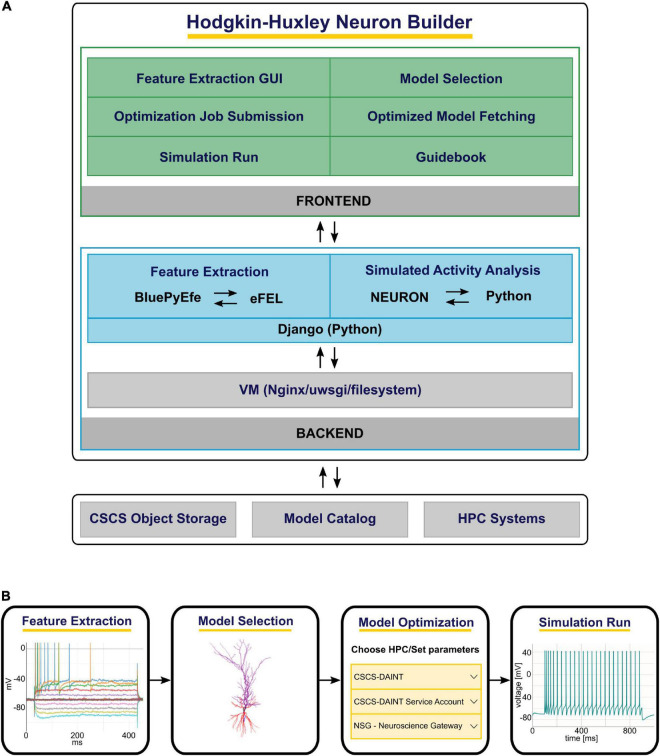
The Hodgkin-Huxley Neuron Builder (HHNB) architecture and workflow. **(A)** From top to bottom: The HHNB frontend allows to interact with the web application in order to extract electrophysiological features from recorded or simulated data, choose a model from the EBRAINS Model Catalog or upload the model’s components, submit the optimization job to an HPC system, fetch the optimized model and run the simulation. The HHNB backend performs the feature extraction, the optimized model analysis and all the data management operations (e.g., files and folders creation, data upload/download). The interaction with the external platforms is guaranteed via direct access (CSCS Object Storage and Model Catalog) or dedicated APIs (HPC Systems). **(B)** The various steps involved in the HHNB workflow. Users are offered flexibility to undertake these steps in the order of their choosing.

The HHNB consists of: (1) A frontend, which provides a point-and-click interface through which the users can execute the various steps of the optimization workflow (e.g., visualize the electrophysiological traces, set the optimization parameters, submit the jobs to the HPC systems), and (2) a backend, where system operations and data processing are performed (e.g., feature extraction, optimized model analysis) while hiding the technical details of the implementation from the users. The HHNB interacts with external platforms, where data and models are stored and computational resources are hosted (i.e., the CSCS Object Storage, the EBRAINS Model Catalog and the HPC Systems) and made available to the user in a transparent manner (see Figure 1A and section “Methods”). The optimization workflow consists of four main steps: (1) Feature extraction/upload; (2) model selection/upload; (3) optimization parameter setting and launch; (4) optimized model simulation (see Figure 1B). In order to provide as much flexibility as possible, the HHNB does not force the users to follow the workflow steps in a given order. For example, users might want to explore (and select) the models available for optimization or visualize the HPC systems at their disposal before performing the feature extraction procedure. Additionally, it is possible to upload one’s own feature and/or model files so as to leverage the HHNB functionalities to perform analysis and optimization on one’s own scientific results (either combined with the datasets available in the HHNB or consistently and fully provided by the users).

**FIGURE 2:**
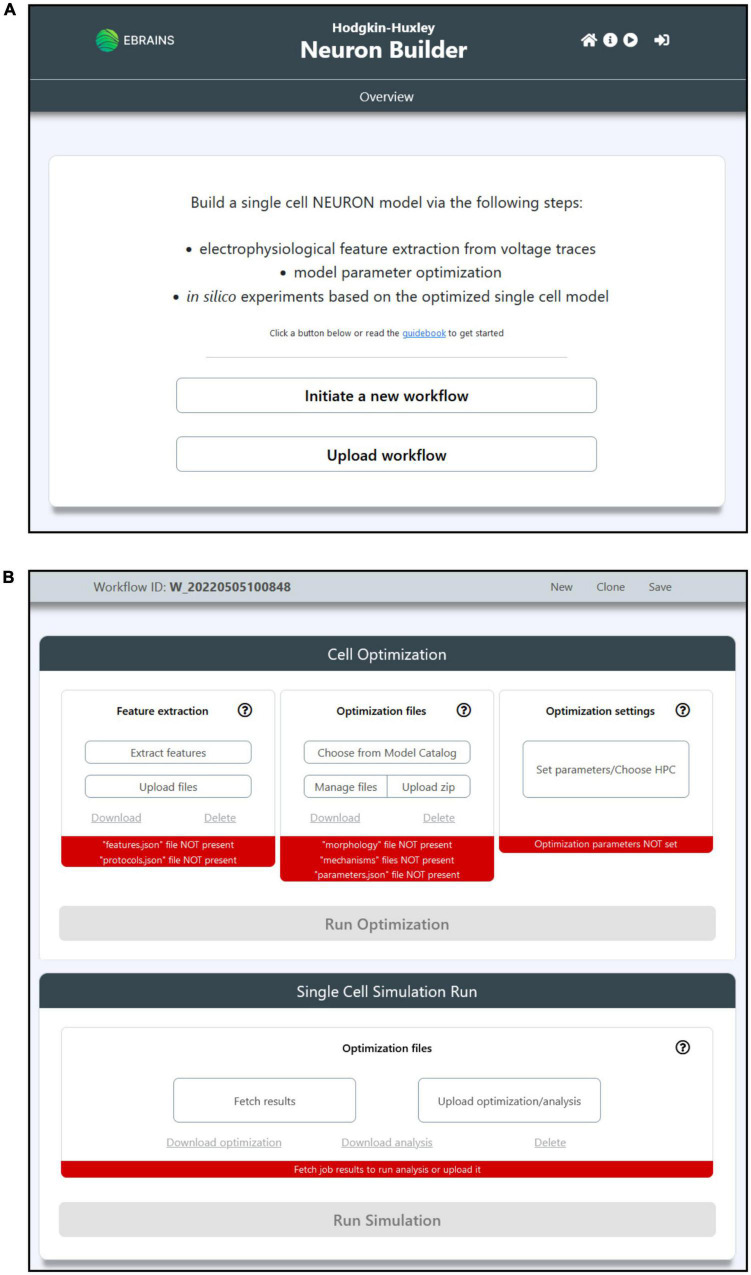
The HHNB GUI. **(A)** The Homepage allows users to initiate a new workflow or upload a previously saved one from their local machine. The header presents, in the top-right section, four icons that allow to (from left to right): (1) Go to the Homepage; (2) open the HHNB Guidebook, which offers detailed documentation for the usage of the HHNB; (3) play the web application video tutorial; (3) login to the EBRAINS authentication system (users must be authenticated to submit jobs to the CSCS HPC system). **(B)** The workflow page presents two panels (from top to bottom): (1) Cell optimization and (2) single cell simulation run. The Cell Optimization panel is divided into three sections that allow to (from left to right): (1) Extract electrophysiological features via the NeuroFeatureExtract (NFE) web application, upload user’s own data, download or delete current feature files; (2) choose a model from the EBRAINS Model Catalog, upload user’s own models previously downloaded from the HHNB, download or delete current model file; (3) choose the HPC system to be used for the model optimization process and set the job and optimization algorithm parameters. The Single Cell Simulation Run panel allows to check the status of submitted jobs and fetch them from the HPC systems.

The HHNB GUI has been designed to be simple and intuitive. The main/overview page allows users to initiate a new workflow or upload one that has been previously saved (the “Save” button is made available once the workflow is initiated); each workflow is uniquely identified via a workflow ID. The main workflow page reflects the context separation between the operations to be performed before and after the model optimization via two panels, the “Cell Optimization” and the “Single Cell Simulation Run” (see Figures 2A,B). The former gives access to dedicated subpages and windows to perform the feature extraction, model selection and optimization parameter configuration; the latter allows to run simulations of the optimized model. In addition to the possibility to upload one’s own feature and model files in their relative sections, the users can also download or delete partial results or selected data (e.g., the extracted feature files or the model chosen to be optimized) and start over with the relevant steps. Finally, the job optimization submission and the simulation run are available (e.g., via the activation of the dedicated buttons) once all the needed operations are completed, as indicated by the red/green indicators at the bottom of the relative panels (see Figure 2B).

### Feature extraction

**FIGURE 3:**
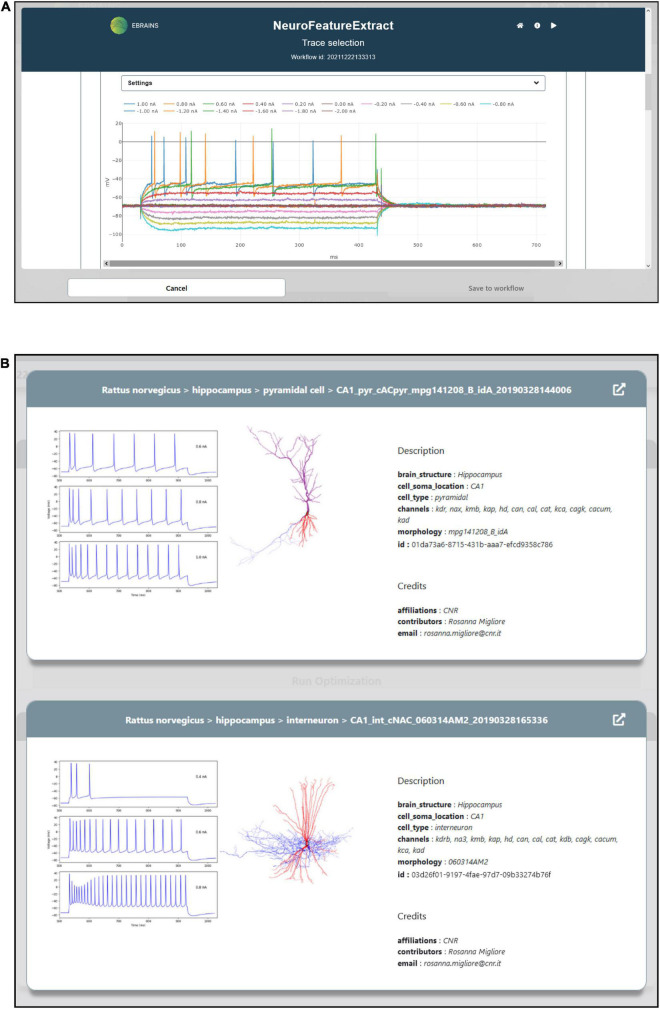
Feature extraction and model choice interface. **(A)** The HHNB integrates, in a dedicated window, the NFE web application that allows users to extract the electrophysiological features of interest against which the single cell neural model will be optimized. **(B)** The model to be optimized can be chosen from a list of models available in the EBRAINS Model Catalog. For each model, a panel is provided where information on the model components and metadata, and representative images of the model morphology and behavior are displayed. In order to access more detailed information about the models, the relative EBRAINS Model Catalog pages can be opened via the top-right arrow button.

To optimize the model parameters and behavior against the observed electrophysiological functioning of the neuronal cell of interest, a feature extraction procedure that allows the extraction of relevant characteristics of neural signals is available in the HHNB. The feature extraction process is enabled through the integration of a web-application, the NFE, that allows visual inspection of electrophysiological traces (see Figure 3A), feature extraction parameter setting, feature selection and results download. The tool has been thoroughly described in Bologna et al. (2021) and is hosted in the same VM as the HHNB, guaranteeing a seamless interaction between the two frameworks (see the “Methods” section). Briefly, the NFE allows to select electrophysiological traces from a dataset of recordings hosted in the EBRAINS KG or contributed by research collaborators and made available via the application GUI. The provided data are labeled according to their cellular and experimental characteristics (e.g., species, brain area, electrical type, stimulus amplitude) and can be explored via a visualization interface that allows one to zoom the traces in and out and selectively choose the signals from which the features will be extracted (the users can also upload and appropriately label their own data). The GUI also allows the feature extraction parameter configuration and the selection of features of interest to be performed before the extraction process is launched. The latter uses the eFEL and BluePyEfe Python libraries that offer advanced functionalities for the data analysis step and the result file formatting (see section “Methods”). The output format is compatible with the BluePyOpt optimizer, and the results files are transparently managed in the creation of the folder structure to be submitted to the HPC system for the optimization process (see section “Model selection”). We suggest that users extract features from homogeneous electrophysiological traces, namely from recordings acquired from cells displaying the same neural behavior. This is because using traces with heterogeneous spiking behaviors, for example those recorded from cells belonging to different electrical types (Markram et al., 2015), might lead to a biophysically implausible model.

### Model selection

A NEURON model optimization consists of the quest for the best set of peak ion channel conductance and passive electrical properties, consistent with the electrophysiological characteristic of the experimentally recorded neuronal voltage traces that the model is aiming to reproduce. HHNB users select an entry from a list of models (see Figure 3B). These have already been optimized in the framework of a research study focusing on the behavior of hippocampal cells (Migliore et al., 2018), via the BluePyOpt optimizer (see section “Methods”) against specific hippocampal neural data.

Using one of these validated models as a starting point and leveraging the target parameter mean and standard deviation values obtained in the feature extraction step, users can run their own optimization to develop a model that better reproduces the selected data. The available models are hosted in and fetched from the EBRAINS Model Catalog (see section “Methods”), which contains, for each model, a detailed description, a download link, the version history and, optionally, information on validation tests undertaken on the model (i.e., how well the model reproduce experimental findings). With respect to the biophysical properties of such models, the properties, which the models are provided with, are, mainly, the membrane passive conductance and the equilibrium potential (defined as *gpas* and *epas* in the NEURON modeling environment, respectively). The peak ion channel conductance has been optimized in the original models (independently for soma, axon, basal and apical dendrites, namely each compartment type of the morphology) since it shapes the electrophysiological properties of the neuron through its variations. More specifically, the ion channels in the models are: 1) a sodium current (*Na*); 2) four different types of potassium currents (*KDR, KA, KM*, and *KD*); 3) three different types of calcium currents (*CaN*, *CaL*, *CaT*), the non-specific *Ih* current, and two different types of Ca-dependent K + currents (*KCa* and *Cagk*). All the compartments containing calcium channels are provided with a calcium extrusion mechanism, featuring a single exponential decay of 100 ms. In general, channels are uniformly distributed in all dendritic compartments with the exception of *KA* and *Ih*, whose density increases with distance from the soma in pyramidal cells. Finally, the electrophysiological features against which the models available to the users were originally optimized, were extracted (via the eFEL library, see section “Methods”) from recordings of individual cells grouped by electrical type (e-type), classified according to their firing patterns (Markram et al., 2015), using the Petilla convention (Ascoli et al., 2008). In the available models, the e-types considered were: (1) cAC (continuous accommodating cells); (2) bAC (bursting accommodating cells); (3) cNAC (continuous non-accommodating cells).

**FIGURE 4:**
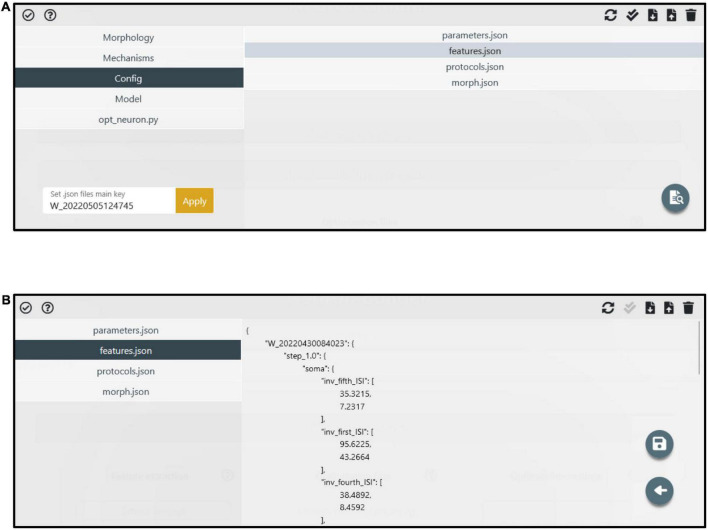
Manage files panel. **(A)** The model components (i.e., files and folders) are accessible through the left menu. The folder content is visible in the right panel after a folder is selected. Via the top-right buttons, users can upload, download, delete and update the folder content. Via the bottom-right button, specific files (e.g., the feature extraction result files) can be edited. **(B)** An example of a file (i.e., *features.json*) in edit mode. A dedicated button (i.e., the floppy disk icon) allows to save the modified file.

Most importantly, while the models available from within the HHNB for further optimization are built according to the described paradigm and properties, the selected model is not necessarily used “as is,” in that the users can modify it—e.g., change the model description in the source file, remove or add model files—thanks to a dedicated panel accessible via the “Manage files” button (see Figure 2B). Provided that the files needed for the execution of the BluePyOpt optimizer are not editable and the same folder structure of the available models is kept, the interface can be used to create the model from scratch, namely, without previous selection of a model from the provided list (see Figures 4A,B).

### Optimization job submission

**FIGURE 5:**
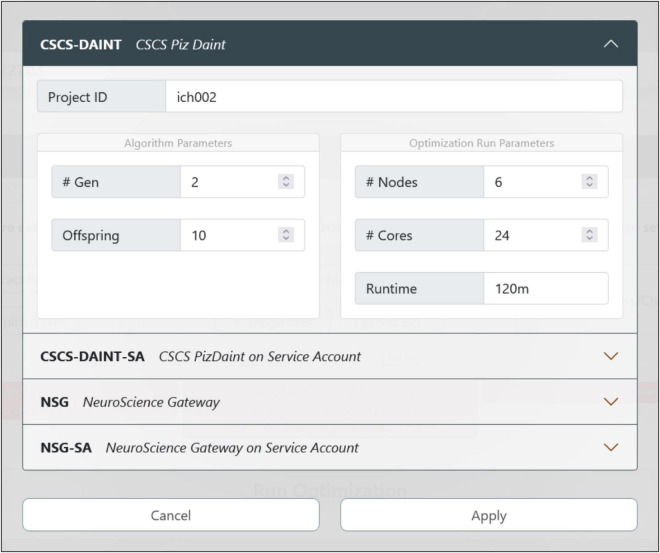
Optimization job configuration. The user can choose the HPC system where the model optimization will be run. There are currently four options available: If the users already have an account (and is part of a project) on the CSCS or NeuroScience Gateway (NSG) supercomputers, they can use their own credentials and quota for the execution of the job. While using NSG requires the insertion of username and password, the CSCS and EBRAINS users’ accounts are automatically mapped together so that no credential insertion is required. In case the users do not have their own account on any of the available HPC systems, they can submit the job via the CSCS or NSG service accounts that only require a registration on the EBRAINS platform (to generate a user token at runtime) and provide a limited quota for job submission.

The HHNB relies on the computational power of HPC systems to carry out the model optimization process. In order to exploit high-performance computing facilities related to the HBP, the users need to be members of an HPC project. In the HBP/EBRAINS framework, users complying with the requirement of having both an EBRAINS account and an HPC project membership on one of the partner platforms have their HPC and HBP identities mapped together. This allows transparent job submission (i.e., without credential requirements) to the HPC systems from the EBRAINS platforms, e.g., the EBRAINS JupyterLab (lab.ebrains.eu) or from web applications that use EBRAINS authentication (as in the case of the HHNB). In the HHNB, we provide an interface where the CSCS-DAINT and the NeuroScienceGateway (NSG) can be chosen as HPC systems for the optimization jobs (see Figure 5; in the case of NSG username and password must be provided).

**FIGURE 6:**
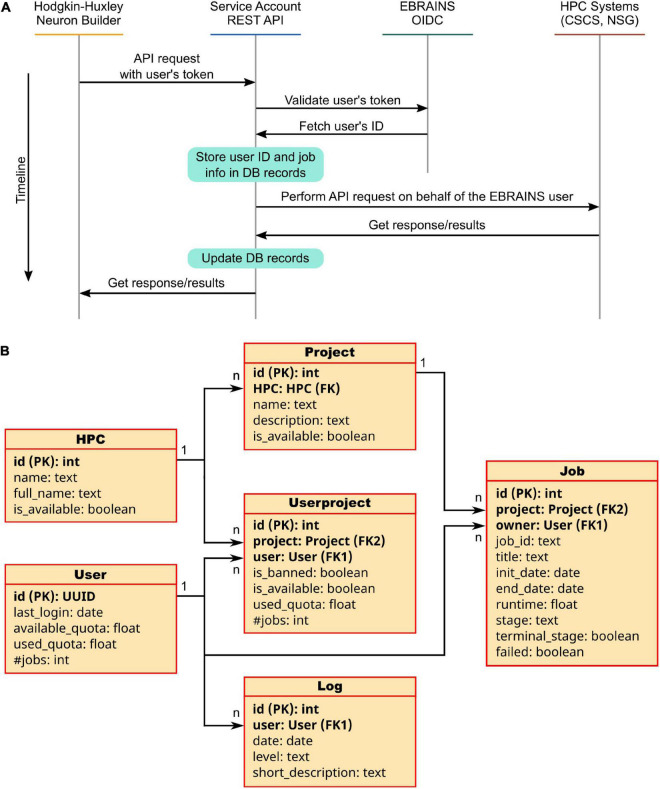
The Service Account Utility. **(A)** Service Account workflow: once logged in to the EBRAINS authentication system, a user token is generated and sent to the Service Account server. Here, the EBRAINS OIDC engine is queried to check the validity of the token. Information on the job to be submitted (e.g., quota, job id, time requested) are stored in the Service Account database. The job is then submitted on behalf of the user to the HPC system. Once the job is completed, the Service Account APIs allow to fetch the results and provide them to the user in a transparent way. **(B)** Architecture of the Service Account database: Six tables are used to keep track of the submitted jobs’ details, users’ quotas and project used. For each table property, the data type is indicated; primary and foreign keys are noted as PK and FK, respectively.

While this authentication (either via token or via credentials) and submission procedure comes with almost no extra-effort for researchers exploiting their own HPC allocation or able to easily get one, it is likely inoperable by prospective users who are not accustomed to nor have the qualification for completing a HPC project submission (e.g., students), do not belong to any research group, or act as independent researchers. To overcome this problem, prone to drastically reduce the number of the HHNB users, we developed a service, the Service Account Utility (see Figure 6 and section “Methods”) that grants any HHNB visitor a limited amount of resources (10,000 node-hours overall by default, but an increase is possible upon request) for computationally intensive job submission. Both the CSCS-DAINT and NSG systems are available to be used via the service account utility and more platforms are being integrated to further extend this functionality.

The job configuration panel provides entries to set both the algorithm and the optimization run parameters. The users can set the maximum number of generations and the number of offspring that the genetic algorithm, implemented in the BluePyOpt optimizer, will adopt while converging toward a solution (see section “Methods”). With respect to the system configuration, the number of nodes and cores of the chosen HPC platform can be set as well as the maximum run time (after which the process is interrupted). For the models presented in Migliore et al. (2018), and available for selection in the HHNB (see section “Model selection”), a typical optimization run had the number of offspring set to 128 and the maximum number of generations set to 60. For a typical optimization on the CSCS HPC system, 6 nodes equipped with 24 CPUs per node were used; the overall optimization time was approximately 15 h. While this number is representative of a sample optimization, it might change, even significantly, depending on the number of parameters to be optimized, the number of features considered and the optimization parameters chosen (e.g., the offspring size). Hence, since the quota deducted from the user’s total amount is the one effectively consumed during the job execution, and not the one requested at submission time, users may wish to set a larger run time for their submissions, so as not to take the risk of having their job interrupted before the optimization has been finalized.

### Optimized model fetching and analysis

**FIGURE 7:**
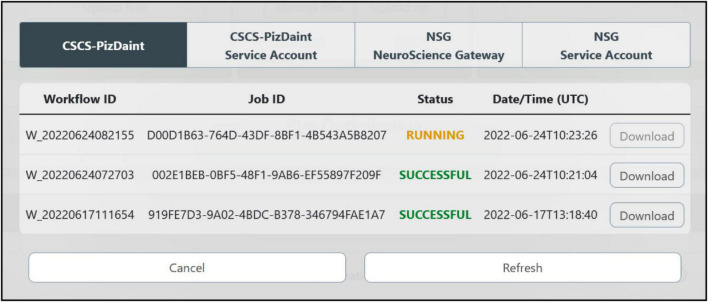
HPC job result fetching. A dedicated window allows the users to check the status of the submitted jobs, grouped by HPC systems, and download them for model analysis and simulation.

The optimization process can last from a few minutes up to several hours, depending on an ensemble of factors (e.g., optimization and HPC system parameters, complexity of the model to be optimized). In order to allow the users to easily check the status of the optimization job and fetch the results once the process has ended, an interactive window is available (see Figure 7). This allows users to visualize the HPC Job ID, the Workflow ID (the unique identifier of the HHNB workflow from which the job was submitted), the status of the job (e.g., queued, running, successful) and the submission date and time. The output of the optimization process, regardless of whether it was successful or not, can be downloaded to the HHNB server first and, successively, to the user’s local machine. Upon success, the optimization output is written in a dedicated folder (called *checkpoints*) that includes a .*pkl* file with the hall of fame (i.e., the ten best individuals, namely the best performing model variants developed during the process) of the optimization and a log reporting the population statistics and the individual genealogy. The NEURON optimized model is stored in a .*hoc* file. After the results have been downloaded, an analysis step is triggered, which generates the electrophysiological traces reproducing the behavior corresponding to the best solution in the hall of fame. The analysis also generates a plot of the objective scores for the best parameters found and a plot displaying the evolution of the scores observed in consecutive generations of the algorithm (see section “Example workflow: Optimization of a hippocampal pyramidal cell model”). All the optimization results and analysis files can be downloaded by the users.

### Model simulation run and registration in the Model Catalog

**FIGURE 8:**
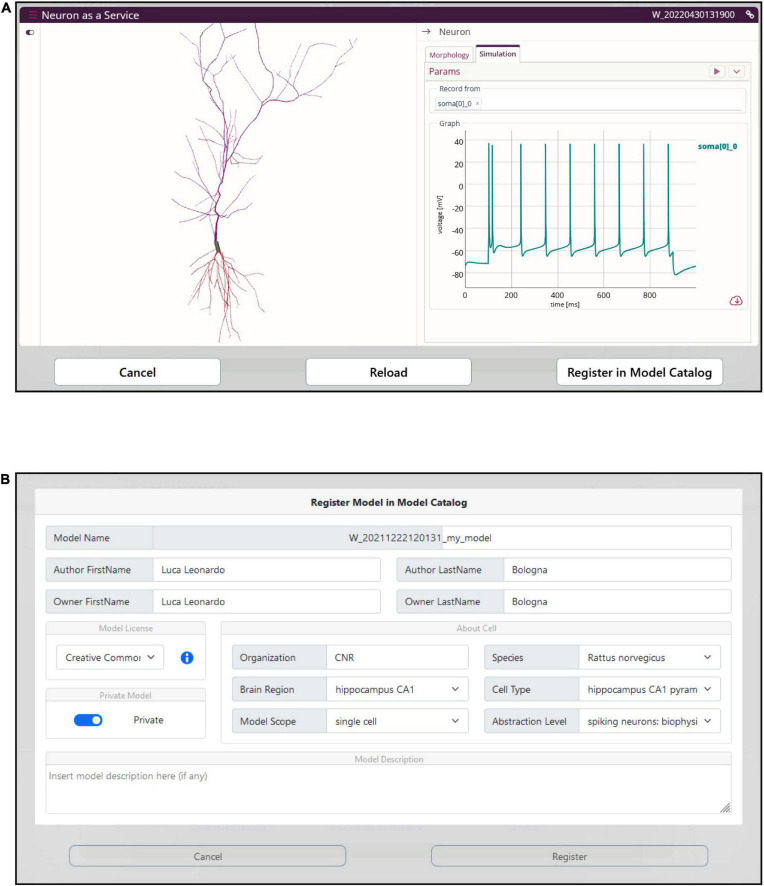
Model simulation. **(A)** The BlueNaas web application is integrated in the HHNB in order to run a model simulation, after the model has been optimized and fetched from the HPC. The GUI allows to set the simulation and stimulation parameters, visualize the cell morphology both in 3D and as a dendrogram, select the recording compartment, visualize the simulated activity, and download the membrane potential value (recorded over the entire simulation) as a .*csv* file. **(B)** A dedicated panel for metadata specification is provided to the users in case they want to register the optimized model in the EBRAINS model catalog.

The optimized model is compiled on the HHNB server and made ready for simulation. In order to provide the users with a fully fledged simulation environment where the simulation parameters can be set and the ongoing simulated activity visualized and downloaded, we embedded the BlueNeuronAsAService (BlueNaas) simulation framework in the HHNB GUI (see Figure 8A). This tool reads the NEURON model (via a dedicated API that transparently enables the model upload) and offers a user-friendly interface that allows to: (1) Visualize the cell morphology both in a 3D interactive image and as a dendrogram; (2) select the neuron compartment from which the neural activity will be recorded; (3) set the stimulus parameter (e.g., temperature, stimulus amplitude and duration, simulation time); (4) visualize the simulated neural activity via an interactive plot; (5) download the simulated activity in a .*csv* file. Should the users be satisfied with how well the optimized model reproduces the electrophysiological recordings (or behaves, in general) they can save it to the EBRAINS Model Catalog by inserting the model details in a dedicated panel (see Figure 8B). The model files (in .*zip* format) are saved in a publicly available EBRAINS Collab and the download link (together with the model properties) is made available in the newly created Model Catalog entry. Upon request, the registered model can be made available in the HHNB as one of the model entries to be chosen for the optimization (see section “Model selection”).

### Example workflow: Optimization of a hippocampal pyramidal cell model

We shall go through an entire single neuron model building workflow, in order to demonstrate the functionalities, flexibility, benefits, ease of use of the HHNB. While the tools and software packages needed for optimizing a single cell NEURON model already exist, and are freely available online (e.g., eFEL, BluePyEfe, BluePyOpt; see sections “Feature extraction, Model selection, Optimization job submission, Optimized model fetching and analysis, Model simulation run and registration in the Model Catalog”), these resources are not natively glued together in an integrated environment and would require the development of *ad hoc* code and visualization frameworks for the completion of a whole workflow. Conversely, thanks to the HHNB, the users will just go through point-and-click actions, thus facilitating the exploration of different data/model coupling, the investigation of optimal HPC resource settings, and the reproducibility of the optimization workflows. Table 2 reports an example data and parameter set that can be adopted to complete a comprehensive and successful (i.e., leading to a model able to faithfully reproduce the experimental data) optimization workflow: (1) selection of two experimental recordings, a subset of traces, and the features to be extracted; (2) choice of the model whose parameters we intended to optimize; (3) configuration of the optimization parameters with respect to both the optimization genetic algorithm and the requested HPC system resources.

**TABLE 2:** List of experimental traces, stimuli, features, model and optimization settings adopted for the optimization workflow example.

Contributor	Species	Structure	Region	Type	Etype	Cell name	Filename
**Thomson’s lab, UCL**	**Rattus-norvegicus**	**Hippocampus**	**ca1**	**Pyramidal-cell**	**Cacpyr**	**95831003**	**95831003**
						95831004	95831004
Stimulus amplitudes selected.							
1 nA, 0.8 nA, 0.6 nA, –0.2 nA, –0.6 nA, –0.8 nA.							
Extracted features.							
“inv_fifth_ISI,” “inv_first_ISI,” “inv_fourth_ISI,” “inv_last_ISI,” “inv_second_ISI,” “inv_third_ISI,” “mean_frequency,” “Spikecount,” “steady_state_voltage,” “voltage_base.”							
Model to be optimized.							
Rattus norvegicus > hippocampus > pyramidal cell > CA1_pyr_cACpyr_mpg141208_B_idA_20190328144006.							
Optimization settings.							
# Gen: 24; Offspring: 10; # Nodes 6; # Cores: 24; Runtime: 2; HPC system: CSCS-DAINT.							

**FIGURE 9:**
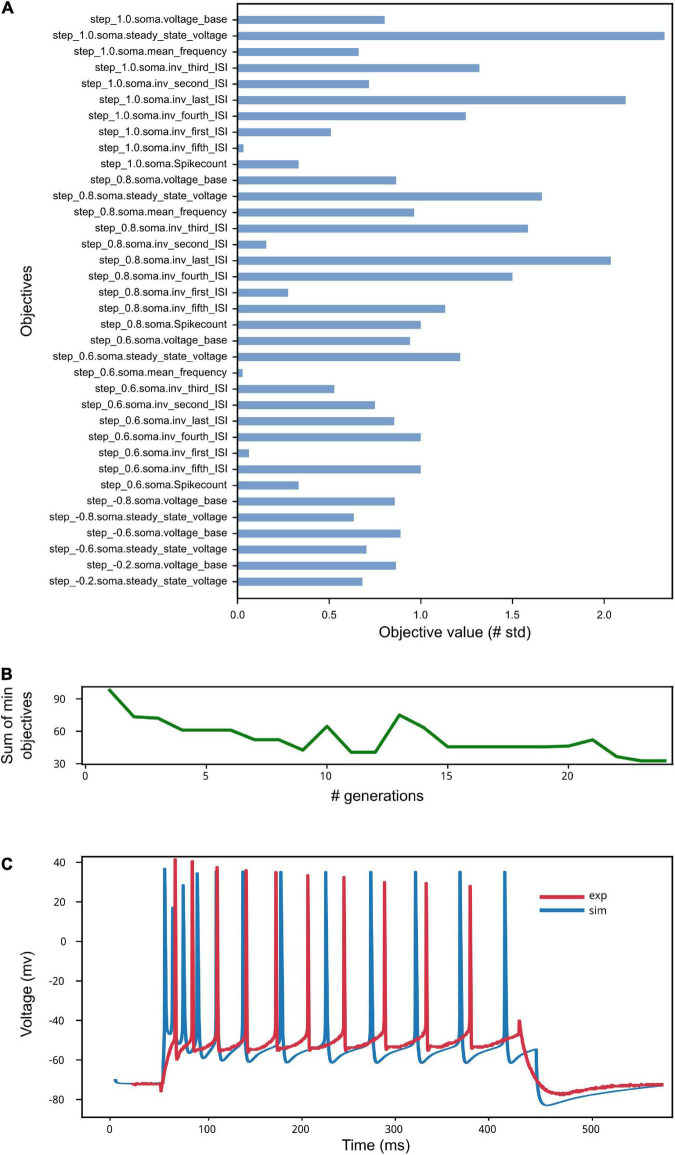
Model Optimization results. **(A)** The objective scores convey information on the deviation of the electrophysiological features computed on the simulated activity from the same features values extracted from *in vitro* experiments and used in the optimization process (see Van Geit et al., 2016). Close-to-zero values have not been reported for ease of visualization. **(B)** The sum of the smallest objectives tends to decrease as the number of generations adopted for the genetic algorithm execution increases. **(C)** Example of simulated model activity recorded at soma location for a 1 nA step stimulus amplitude, plotted against one of the experimental traces selected for the extraction of the features used for the model optimization. The spike count and the spike timing are comparable as well as the voltage base value. Given that the experimental behavior that the model aims to capture is obtained against the average feature values extracted from multiple recordings, individual simulated and experimental traces will present variable differences, depending on the extent of success of the optimization process.

The results of the optimization are shown in Figure 9. The low standard deviation of the observed objective values (see Figure 9A) indicates that the optimization process was able to capture the average feature values extracted from the recorded activity. This result is reached thanks to the genetic algorithm implemented by the BluePyOpt optimizer, which refines the parameter values over several generations (see Figure 9B). As a result of an effective optimization, the simulated neural activity can reproduce the average experimental behavior (e.g., in terms of number of spikes, voltage base value, spike timing) (see Figure 9C). Depending on the variability of the experimental data, the features of interest and the model to be optimized, a fine tuning of the optimization process settings might be required, for example in terms of number of generations and offspring to be set.

## Discussion

In the framework of the HBP (Amunts et al., 2016) and the EBRAINS research infrastructure,^16^ we have developed an online resource for the optimization of biophysically detailed single cell NEURON models (Hines and Carnevale, 1997) based on experimental results. The HHNB allows users, via a user-friendly GUI, to go through an entire model building workflow that includes: (1) The extraction of electrophysiological features from a dataset of traces or from recordings provided by the user; (2) the choice of a model to be optimized or the upload of well-described model files built by the user; (3) the optimization of the model based on the extracted features; the interface allows the configuration of the parameters related to the genetic algorithm underlying the optimization as well as the specification of the HPC system resources to be used for running the process; (4) the simulation of the optimized model via a graphical interactive interface, which offers functionalities for setting simulation and stimulus parameters as well as visualizing and downloading the simulated neural activity.

The HHNB adds unique features to the ecosystem of tools and platforms for neural data analysis and simulation. Thanks to the integration of several software packages and online web applications in a user-friendly online environment, the HHNB offers a plethora of functionalities that usually require distinct workflows and/or stages of application execution, software installation and data homogenization. The feature extraction is run using the NFE resource (Bologna et al., 2021), embedded in the HHNB and residing in the same VM as the HHNB so as to allow a seamless communication between the two web applications and faster data and result file management. Data available in the NFE are hosted in the EBRAINS KG or in public EBRAINS data containers. The NFE exploits the BluePyEfe and the eFEL Python libraries (see section “Methods”) by hiding from the user the technical details of configuration file writing and data management (as required when both tools are run in a standalone manner on a local machine) and exposing a user-friendly point-and-click interface, instead. The models available for the optimization are fetched from the EBRAINS Model Catalog (see Table 1), which provides details and links related to the modeling work carried out in the framework of the HBP/EBRAINS research infrastructure as well as results concerning the validation of models against experimental observations (Sáray et al., 2021). A tight integration is also in place between the HHNB and the HPC systems available for job optimization: via the UNICORE Python library or dedicated APIs for the interaction with the CSCS-DAINT system and the NSG, respectively, and using an intuitive web interface, the HHNB seamlessly allows to authenticate to the remote platforms, configure the job execution files and fetch the optimization results. With respect to HPC use, an additional service has been developed to guarantee the availability of the HHNB to as large an audience as possible: the Service Account Utility allows the optimization jobs to be run (and the workflow to be completed) by users who neither have credentials on any of the available HPC systems nor are part of any HPC project. The only requirement for using such functionality is to have an EBRAINS account (registration is free at https://ebrains.eu/register, and only requires an institutional email address). Finally, the BlueNaaS simulation tool has been embedded in the HHNB interface using a dedicated API, specifically developed for this purpose, that allows to transparently upload the optimized model to the BlueNaaS server, display the model morphology, set the execution and stimulus parameters, and run the simulation. Via the same interface, users can also register the model in the EBRAINS Model Catalog and make it available to the scientific community.

While the HHNB already offers a complete environment for electrophysiological data analysis, model optimization and simulation, a number of improvements and upgrades are planned in order to provide the users with a better experience and further promote collaborative research. The interaction with the EBRAINS KG is being made tighter: we plan to develop an interface that automatically updates the list of available electrophysiological traces and NEURON models suitable for the optimization workflow as soon as they are available in the KG. A further improvement will leverage the EBRAINS provenance engine, currently under development, to keep track of the data recordings used, the parameters adopted for the feature extraction and the model optimization, the model files chosen or uploaded by the users and the HPC resources exploited. This will allow to flawlessly reproduce users’ workflows for data comparison and validation or further analysis. Finally, we plan to strengthen the collaborative aspect of the HHNB by creating a seamless integration with the EBRAINS Collaboratory environment.^17^ The EBRAINS Collaboratory provides a framework to create and share documents, tools, code and applications via dedicated workspaces called *Collabs*, which are individually linked to a data drive^18^ that can be used as a common repository. In order to provide the scientific community with a resource able to foster research collaboration, we plan to leverage the storage space of the EBRAINS Collaboratory and the services it offers for saving and sharing the HHNB relevant data and metadata files, to allow partners and collaborator to visualize, download, finalize or modify existing or newly created HHNB workflows.

## Data availability statement

Publicly available datasets were analyzed in this study. This data can be found here: https://hbp-bsp-hhnb.cineca.it/hh-neuron-builder/.

## Author contributions

LB wrote thes first draft. MM and LB contributed to the conception and design of the study. LB, RS, and CL contributed to the implementation of the HHNB web application. SA and AD contributed to the design and implementation of the EBRAINS Model Catalog and to its integration with the HHNB. GI and J-DC contributed to the design and implementation of the BlueNaas and to its integration with the HHNB. All authors contributed to the manuscript revision and approved the submitted version.

## Footnotes

1
www.nsgportal.org

2
cscs.ch

3
juelich.de

4
cineca.it

5
dandiarchive.org

6
zenodo.org

7
datadryad.org

8
figshare.com

9
hippocampome.org

10
neuromorpho.org

11
alleninstitute.org

12
https://www.djangoproject.com/

13
https://www.openstack.org/

14
https://spack.io

15
https://www.django-rest-framework.org/

16
https://ebrains.eu/

17
https://wiki.ebrains.eu

18
https://drive.ebrains.eu
